# Using Clinical Trials as Agents of Transformation in Populations Burdened by Disparities

**DOI:** 10.1093/geroni/igab046.491

**Published:** 2021-12-17

**Authors:** Daniel Jimenez, Giyeon Kim

## Abstract

Older adults from racial/ethnic backgrounds as well as those from rural areas experience a disproportionate burden of physical and mental health risk factors. Given the prevalence of comorbid physical and mental health conditions in later life, the inadequacies of current treatment approaches for averting years living with disability, the disparities in access to the health care delivery system (including mental health care), and the workforce shortages to meet the mental and physical health needs of racial/ethnic and rural populations, development and testing of innovative strategies to address these disparities are of great public health significance and have the potential to change practice. This session will illustrate how four different interventions are being used to address mental and physical health needs in Latino and rural-dwelling older adults with the goal of reducing and ultimately eliminating disparities in these populations. Particular attention will be paid to the use of non-traditional interventions (e.g. social support, health promotion, technology). Results of clinical research studies will be presented alongside clinical case presentations. This integrated focus highlights the importance of adapting research interventions to real-world clinical settings.

